# Prediction and verification of microRNA targets by MovingTargets, a highly adaptable prediction method

**DOI:** 10.1186/1471-2164-6-88

**Published:** 2005-06-08

**Authors:** Craig Burgler, Paul M Macdonald

**Affiliations:** 1 Section of Molecular Cell and Developmental Biology, Institute for Cell and Molecular Biology, The University of Texas at Austin, 1 University Station A-4800, Austin, TX 78712-0159, USA

## Abstract

**Background:**

MicroRNAs (miRNAs) mediate a form of translational regulation in animals. Hundreds of animal miRNAs have been identified, but only a few of their targets are known. Prediction of miRNA targets for translational regulation is challenging, since the interaction with the target mRNA usually occurs via incomplete and interrupted base pairing. Moreover, the rules that govern such interactions are incompletely defined.

**Results:**

MovingTargets is a software program that allows a researcher to predict a set of miRNA targets that satisfy an adjustable set of biological constraints. We used MovingTargets to identify a high-likelihood set of 83 miRNA targets in Drosophila, all of which adhere to strict biological constraints. We tested and verified 3 of these predictions in cultured cells, including a target for the Drosophila *let-7 *homolog. In addition, we utilized the flexibility of MovingTargets by relaxing the biological constraints to identify and validate miRNAs targeting *tramtrack*, a gene also known to be subject to translational control dependent on the RNA binding protein Musashi.

**Conclusion:**

MovingTargets is a flexible tool for the accurate prediction of miRNA targets in Drosophila. MovingTargets can be used to conduct a genome-wide search of miRNA targets using all Drosophila miRNAs and potential targets, or it can be used to conduct a focused search for miRNAs targeting a specific gene. In addition, the values for a set of biological constraints used to define a miRNA target are adjustable, allowing the software to incorporate the rules used to characterize a miRNA target as these rules are experimentally determined and interpreted.

## Background

MicroRNAs (miRNAs) are an abundant evolutionarily conserved class of small (~22 nts) RNAs which play a substantial gene regulatory role in plants and animals [1]. The first miRNA discovered, *lin-4*, was identified in a genetic screen focused on identifying genes involved in the heterochronic pathway in C. elegans [2]. The 22 nt *lin-4 *transcript temporally negatively regulates translation of *lin-14*, apparently through antisense RNA-RNA interaction between the *lin-4 *transcript and multiple regions in the *lin-14 *3' UTR. Seven years later a second small RNA, *let-7*, was found, and it too acts in the heterochronic pathway in C. elegans [3]. *let-7 *represses translation of *lin-41 *in a temporally dependent manner, also through targeting complementary regions in the 3' UTR of the regulated gene [4].

*let-7 *transcripts are found in all bilaterians tested [5]. This discovery led to the understanding that miRNA-mediated regulation may be a general phenomenon. Several hundred miRNAs have since been identified in a variety of plants and animals through cloning and computational methods, including 78 miRNAs in Drosophila [6]. Many of these miRNAs are expressed in a temporal or tissue-specific dependent manner [1].

miRNAs in animals usually act to repress translation of their target genes through imperfect hybridization to complementary sites in target 3' UTRs [2,7,8]. This translational repression occurs post-initiation, since miRNA-induced gene silencing does not change the abundance or polysome profile of target mRNA, at least in the examples tested [9,10]. This is in contrast to RNAi, in which short RNAs called siRNAs are usually perfectly complementary to their target mRNA and result in its degradation [11-14]. A miRNA directed against a perfectly complementary 3' UTR target site also results in mRNA degradation [15,16], indicating that a miRNA can function in the RNAi pathway given a perfectly complementary target site. miRNAs are produced from a larger transcript through stepwise processing by ribonuclease III-like endonucleases in the nucleus and cytoplasm [17-19]. Following maturation, miRNAs reside in a miRNA ribonucleoprotein complex (miRNP) which shares many similarities to the RNA-induced silencing complex (RISC) involved in RNAi [1,16,20-22].

While many animal miRNAs have been identified, only a few have a known function or target [2,23-28]. Incomplete base pairing of miRNA to target causes inherent difficulty in the prediction of miRNA targets due to the high levels of noise involved in any simple alignment of miRNAs to 3' UTRs. In addition, the very few experimentally derived miRNA/target pairs provide limited biological information needed to define the necessary and sufficient characteristics for a miRNA/target pair. Therefore, miRNA target prediction programs for which the selection parameters can easily be adjusted based on current interpretation of miRNA/target constraints, and on newly discovered rules governing miRNA/target interactions, are a valuable resource to the research community.

## Implementation

Our bioinformatics approach to identifying miRNA targets includes two steps: the creation of a database of potential targets, and screening all possible miRNA/target pairs for adherence to constraints suggested by analysis of the known miRNA/target interactions.

### Potential miRNA target database

The selection of sequences for the database was guided by progress in understanding the actions and features of miRNAs and their targets. All known animal miRNAs appear to target regions in the 3' UTRs of mRNAs [2,23-28], and so the database was limited to 3' UTR sequences.

miRNAs are highly conserved, particularly in closely related species [1,5], and 68 of the 78 known D. melanogaster miRNAs are identical to their predicted counterparts from D. pseudoobscura. The constraint on miRNA evolution is thought to be a consequence of their interaction with multiple targets, thereby restricting the rate of change of both the miRNA and its targets [29]. In addition, miRNA targets that have been experimentally derived are very highly conserved in other species [1,5,7,24,27]. Therefore, the database was further limited to highly conserved regions of 3' UTRs.

Finally, the majority of experimentally derived miRNA targets contain multiple predicted target sites[1,2,23-28]. Our approach to allow detection of multiple targets in a single 3' UTR was to fragment the database sequences into segments no longer than 50 nt, each of which is tested for target sites. For conserved 3' UTR segments longer than 50 nt, overlapping 50 nt segments with end points differing by 5 nt were added to the database (i.e. a 100 nt sequence would be fragmented into 11 overlapping 50 nt segments). We chose 50 nt for the maximum target site length since all predicted target sites of known miRNA targets are less than this size [2,23-28].

The Berkeley Genome Pipeline [30,31] was accessed to obtain all 3' UTR sequences which are at least 80% conserved between D. melanogaster and D. pseudoobscura, and are at least 12 nt long [the smallest predicted target site of known miRNA targets that we are aware of is 13 nts in length [2]]. The D. pseudoobscura genome sequence is largely known, but the last stages of sequence finishing (to allow assembly of the final sequence from the large number of shorter contigs) and annotation are not complete. Thus some regions of the D. pseudoobscura sequence that are highly conserved with D. melanogaster 3' UTRs have not been definitively linked to a coding region. Nevertheless, these conserved sequences were included to ensure that the largest fraction of potential miRNA target sites would be evaluated in the prediction process. Using D. melanogaster genome annotation release 3.1 (BDGP) we obtained 14,287 annotated 3' UTRs. Of these, 6702 contained unique segments, at least 12 nt in length and 80% conserved with D. pseudoobscura DNA, that were included in the database. The 6702 database entries correspond to 6399 different genes, with some genes represented more than once because of alternate splicing.

### Biological miRNA target constraints

The MovingTargets algorithm applies a set of five biological constraints to all possible alignments of each miRNA with the miRNA target database sequences, producing a set of predicted targets. The user sets values for the constraints. This adjustable algorithm facilitates focused searches with individual mRNAs, where experimental evidence may suggest that miRNA-dependent regulation exists.

1) Number of target sites in the mRNA. For most of the known miRNA/target mRNA pairs the miRNA is predicted or known to interact with multiple sites within the 3' UTR [23-28]. Furthermore, there is experimental evidence of a synergistic effect between multiple miRNP complexes associated with a single mRNA [21], suggesting that multiple target sites may allow for rapid translational control [32].

2) Strength of miRNA-mRNA hybridization. The specific interaction of a miRNA with a target mRNA involves base pairing [4], and it is reasonable to assume that target site occupancy will be positively correlated with the strength of the base pairing [22-28]. We therefore rank potential miRNA/target interactions according to the strength of hybridization between the miRNA and its target site, as measured by the predicted free energy of binding. These predictions are made using M. Zuker's DINAMelt Server software [33] which was expressly designed for evaluating the interactions of short RNAs and thus offers advantages over the commonly used alternatives, mFold [34] and RNAfold [35].

3) Number of consecutive base pairs involving the 5' part of the miRNA. There is suggestive evidence that miRNA/target interactions require a series of consecutive base pairs between the 5' part of the miRNA and the target [7,23-28,36-38]. Of the experimentally validated animal miRNA targets [2,23-28], 19 of the 24 predicted miRNA/mRNA interactions have 6 or more consecutive base pairs within the first 8 nucleotides of the miRNA; 10 of these interactions have perfect complementarity in this region. This is contrasted with only 5 of the 24 predicted miRNA/mRNA interactions having 6 or more consecutive base pairs at the miRNA 3' end. Note that for almost all examples of mRNAs known to be regulated by miRNAs, the specific target sites in the mRNA (identified as regions with significant complementarity to the miRNA) have not been individually tested and verified.

Additional evidence comes from mutational analysis of miRNAs and their targets. The ability of miR-30 to repress translation of an artificial target in cultured human cells is eliminated by a mutation in the target mRNA that disrupts a single base pair in the middle of the 5' region of the miRNA, while a mutation in the target mRNA disrupting base pairing in the 3' part of the miRNA retains about 60% of the repressive activity [22]. We used the DINAMelt Server to predict the effect of both mutations on hybridization strength, and found that the inactivating mutation had a more modest effect than the weak mutation. These results argue that the important aspect of the interaction disrupted by the first mutation was the consecutive series of base pairs at the 5' end of the miRNA, rather than the strength of the interaction as measured by thermodynamic stability considerations alone. A mutation in the 5' region of the *let-7 *miRNA eliminates repression of *lin-41 *mRNA *in vivo*, but also reduces the level of the mature miRNA, making it difficult to conclude why it is ineffective [3].

4) Total number of miRNA 5' nucleotides involved in base pairing to the target. For mRNAs shown to be miRNA targets, all of the 24 predicted miRNA/mRNA interactions have 6 or more total base pairs within the first 8 nucleotides of the miRNA; 21 of these interactions have at least 7 base pairs. This is contrasted with only 11 of the 24 predicted miRNA/mRNA interactions having 6 or more total base pairs at the miRNA 3' end [23-28]. In addition, there is more stringent sequence conservation in the 5' end of homologous miRNAs than in the 3' end [36].

5) Number of nucleotides in the miRNA 5' region involved in G:U base pairs. Predicted miRNA/target interactions of known miRNA targets have at most one (6 out of the 24 predicted miRNA/mRNA interactions) and usually no G:U base pairs in the miRNA 5' region. In contrast, despite having fewer overall base pairs in the miRNA 3' region, 9 out of the 24 predicted miRNA/target interactions have more than 1 G:U base pair in the miRNA 3' region, and 8 of the 24 have 1 G:U base pair in this region [23-28]. Thus, canonical base pairing appears to be favored over G:U base pairing in the miRNA 5' region.

Subsequent to development of the MovingTargets algorithm an extensive study of the rules of miRNA/target interactions was published [39]. The results emphasize the importance of the latter three constraints described above.

## Methods

### DNA constructs

Reporter plasmids were constructed by cloning the 3'UTR of each target gene into the BamHI/XbaI site of *luk-ttkUTR *[40]. *luc/tramtrack *is the *luk-ttkUTR *plasmid. For *luc/CrebA*, the *CrebA *3'UTR was amplified by PCR from genomic DNA (all sequence coordinates are from Release 3 from FlyBase, ): 3L:15500154-15502103. For *luc/ab*, the *ab *3'UTR was PCR amplified from genomic DNA: 2L:11248260-11249979. For *luc/Eip74EF*, the *Eip74EF *3'UTR beginning at position 29 was cloned from *pBSE74AcDNA*, a gift from Carl Thummel. The control plasmid for monitoring transfection efficiency is *MT-RLuc *[40].

miRNA plasmids were constructed by cloning a DNA segment containing the predicted primary transcript of each miRNA into the BamHI/EcoRI site of *ActMSI *[40]. Each primary transcript was amplified by PCR from genomic DNA to generate fragments with the following sequence coordinates: *let-7*: 2L:18450072-18450291; *mir-92b*: 3R:21466427-21466673; *mir-312: *2R:15647675-15647897; *mir-34: *3R:5926642-5926792. In each case, BamHI and EcoRI sites were introduced at the 5' and 3' ends, respectively, by PCR.

### Targeting Assay

S2 cells were transfected with 2 μg of microRNA plasmid (for non-control samples), 50 ng reporter plasmid, and 10 ng control plasmid. For each transfection, 0.5 mL Schneider's Drosophila Medium (Gibco) containing indicated plasmids and 0.5 mL Schneider's Drosophila Medium containing 5 μL Cellfectin (Invitrogen) were mixed gently and incubated at RT for 15–45 minutes. 2 × 10^6 ^cells were centrifuged for 5 minutes at 1000 g, aspirated, resuspended in the DNA-lipid mix described above, and transferred to a 35 mm well of a 6-well plate. Cells were incubated at 25°C for the remainder of the transfection. After 4–5 hours, 0.5 mL of Schneider's Drosophila Medium containing 30% Fetal Bovine Serum (Gibco) was added to each well. The following day, 2 mL of complete growth medium [Schneider's Drosophila Medium with 10% FBS and 1% Pen/Strep (Gibco)] was added to each well. Between 38 and 45 hours after transfection, the reporter and control plasmids were induced by adding 3.5 μL of 700 mM CuSO4. After 6–6.5 hrs, cells were harvested, lysed, and assayed for reporter and control luciferase expression using the Dual-Luciferase Reporter Assay system (Promega). Each transfection was carried out at least 5 times.

### Potential miRNA Target Database

D. melanogaster 3'UTRs were obtained from the Berkeley Drosophila Genome Project , Drosophila Release 3.1 Annotations. Conserved D. pseudoobscura sequences were obtained from the Berkeley Genome Pipeline using the following parameters: minimum conservation width = 1; calculation window = 20; minimum conservation = 80%. We used the 7-8-03 AVID alignment for determining conserved regions [30,31]

### miRNA sequences

D. melanogaster miRNA sequences were obtained from The microRNA Registry [6].

## Results

In this initial use of MovingTargets we set stringent values for all of the adjustable biological constraints to produce a high likelihood set of miRNA target predictions. The following values were used: minimum of 3 target sites; maximum free energy of hybridization of -15 kcal/mole at room temperature (22°C) for each target site; minimum 7 out of 8 consecutive 5' miRNA nt matches; maximum of 1 G:U base pair in the miRNA 5' region. The high-likelihood set of miRNA target predictions corresponding to these strict biological constraints, generated from analysis of all 78 miRNAs and the full database of 6399 potential targets containing conserved 3' UTR sequences, is given in Table 1. Given the strict constraints, this group will not contain all miRNA/target pairs (and includes no predictions for a subset of the known miRNAs).

**Table 1:** microRNA/target pairs predicted by MovingTargets using the following strict targeting criteria: minimum 3 target sites, maximum dG of microRNA/target hybridization of -15 kcal/mole at RT for each target site, minimum 7 out of 8 consecutive base pairs in microRNA 5' end, maximum 1 G:U base pair in microRNA 5' region. Transcription factor targets are listed first followed by neural targets. Gene function and biological process are as given by FlyBase , March 2004.

Target	miRNA	# target sites	dG of miRNA-mRNA hybrid (kcal/mole)	Molecular Function	Biological process
*CrebA*	*mir-92b* *mir-312*	3 4	-16, -24, -19 -16, -20, -17, -22	transcription factor	salivary gland development
*fkh*	*mir-315*	3	-18, -16, -17	transcription factor	salivary gland morphogenesis
*Eip74EF*	*mir-34*	3	-25, -27, -21	transcription factor	mesoderm development
*Eip93F*^A^	*mir-280*^A^	3	-21, -15, -16	transcription factor	autophagy, induction of apoptosis by hormones
*pros*	*mir-34*	3	-18, -17, -18	transcription factor	ectoderm development
*zfh1*	*mir-5*	3	-17, -15, -15	transcription factor	ectoderm development, mesoderm development
*zfh2*	*mir-276a-3*	3	-16, -15, -17	transcription factor, RNA binding	ectoderm development
*SoxN*	*mir-34* *mir-309*	3 3	-23, -24, -16 -19, -16, -20	transcription factor, DNA bending	ectoderm development, visual perception
*HLHm5*	*mir-7*	3	-24, -24, -26	transcription factor	ectoderm development, cell proliferation
CG32527^B^	*mir-34*	3	-25, -24, -21	multiple (including transcription factor)	unknown
*ab*	*let-7*	5	-20, -25, -17, -17, -19	transcription factor	transmission of nerve impulse, sex determination
*sbb*	*mir-33*	3	-16, -17, -19	transcription factor	axon guidance, axon target recognition, larval walking behavior
*nerfin-1*	*mir-279* *mir-286*	3 3	-19, -17, -24 -19, -16, -27	transcription factor	neuronal lineage restriction
*Syn*	*mir-92b*	3	-19, -20, -15	unknown	neurotransmitter secretion, synaptic vesicle exocytois
*synaptogyrin*	*mir-313*	3	-17, -16, -15	unknown	synaptic vesicle exocytosis, regulation of calcium ion dependent exocytosis
*Pkc98E*	*mir-210*	3	-15, -15, -15	multiple	multiple (including neural processes)
*nAcRbeta-96A*	*mir-210*	3	-19, -18, -23	nicotinic acetylcholine-activated cation-selective channel activity, acetylcholine receptor activity	multiple (including neural processes)
*W*^A^	*bantam* *mir-280*^A^	4 3	-21, -16, -27, -17 -17, -19, -15	unknown	induction of apoptosis, programmed cell death
*kel*	*mir-310* *mir-311* *mir-312*	3 4 4	-20, -19, -22 -17, -21, -19, -15 -16, -18, -20, -17	actin binding	apoptosis, ovarian ring canal formation
*RhoGEF2*	*mir-9c*	3	-16, -16, -18	Rho guanyl-nucleotide exchange factor activity, diacylglycerol binding	multiple
*adat*	*mir-3* *mir-309* *mir-318*	3 3 3	-28, -17, -19 -23, -16, -15 -26, -15, -20	tRNA specific adenosine deaminase activity	purine base metabolism
*bru-2*	*mir-9a* *mir-9c*	3 3	-19, -17, -16 -19, -15, -16	RNA binding	mRNA processing, protein metabolism
CG32062^A^	*mir-12* *mir-280*^A^	4 3	-19, -17, -23, -15 -16, -16, -19	RNA binding	unknown
*fus*	*mir-303*	3	-16, -15, -16	RNA binding	epidermal growth factor receptor signaling pathway
*mbl*^A^	*mir-280*^A^	3	-17, -17, -21	RNA binding, DNA binding	mesoderm development
*Asph*	*mir-9b*	3	-21, -19, -16	peptide-aspartate beta-dioxygenase activity	transmembrane receptor protein tyrosine kinase signaling pathway
*CG32429*	*mir-33*	3	-17, -17, -18	unknown	unknown
*lmg*	*mir-34*	3	-27, -20, -22	unknown	mitotic anaphase
*wb*	*mir-34*	4	-22, -22, -19, -23	binding, structural molecule activity	cell-cell adhesion, cell-matrix adhesion, signal transduction
*Cbl*	*mir-34*	3	-22, -17, -22	ligase activity	cellular defense response
*Pdi*	*mir-34* *mir-263b* *mir-305* *mir-316*	4 3 4 3	-26, -23, -30, -20 -18, -15, -17 -24, -18, -19, -22 -21, -16, -21	protein disulfide isomerase activity	protein folding, protein modification
CG31637	*mir-92b*	3	-17, -18, -17	sulfotransferase activity	carbohydrate metabolism
CG3689	*mir-210*	3	-20, -15, -17	pre-mRNA splicing factor activity	mRNA cleavage, nuclear mRNA splicing, via spliceosome
CG8475	*mir-263b*	3	-18, -17, -21	kinase activator activity, phosphorylase kinase regulator activity	glycogen metabolism
*didum*	*mir-276a-3*	3	-18, -16, -15	multiple	multiple
*Ggamma1*	*mir-277*	3	-19, -16, -15	heterotrimeric G-protein GTPase activity	G-protein coupled receptor protein signaling pathway
CG1441	*mir-278*	3	-30, -22, -26	oxidoreductase activity	unknown
*Rh6*	*mir-278*	3	-19, -22, -20	G-protein coupled photoreceptor activity	phototransduction, visual perception, sensory perception
CG31163^A^	*mir-289*^A^	3	-17, -15, -17	SH3/SH2 adaptor protein activity	unknown
CG18854	*mir-306-3*	4	-27, -19, -24, -21	inositol-triphosphate 3-kinase activity	unknown
CG7908	*mir-309*	3	-17, -19, -22	zinc ion binding, metalloendopeptidase avtivity	cell surface receptor linked signal transduction, proteolysis and petidolysis
CG14507	*mir-276a-5* *mir-276b-5*	3 3	-24, -20, -21 -24, -20, -21	phospholipase A2 activity	unknown
CG33085^A,B^	*mir-184-3* *mir-284*^A^	3 3	-21, -22, -16 -23, -25, -17	argininosuccinate lyase activity	unknown
CG32316^B^	*mir-184-3* *mir-278*	3 3	-20, -22, -16 -18, -20, -23	oxoglutarate dehydrogenase (succinyl-transferring) activity	tricarboxylic acid cycle
CG32912^B^	*mir-279*	3	-15, -21, -16	peptidoglycan recognition activity	immune response
CG33047^A,B^	*mir-133* *mir-284*^A^	3 3	-19, -17, -19 -27, -17, -15	alpha-L-fucosidase activity	O-glycoside catabolism, fucose metabolism
CG33075^B^	*mir-306-3*	3	-26, -20, -27	carrier activity	transport
CG32956^B^	*mir-9a* *mir-9b* *mir-9c* *mir-33*	5 5 5 3	-17, -19, -18, -21, -18 -17, -20, -16, -20, -17 -17, -22, -18, -20, -18 -19, -23, -18	multiple	multiple
CG33038^B^	*mir-9a* *mir-9b* *mir-9c*	4 5 4	-20, -17, -16, -18 -18, -19, -17, -16, -16 -21, -17, -17, -19	multiple	heparan sulfate proteoglycan biosynthesis, proton transport
CG32791	*mir-31a*	3	-15, -17, -15	unknown	multiple
*tinc*	*mir-34*	3	-22, -23, -33	unknown	unknown
CG9932	*mir-263b*	4	-16, -21, -20, -21	unknown	unknown
CG30389	*mir-14* *mir-34*	3 3	-17, -16, -25 -28, -21, -24	unknown	unknown
CG3975	*mir-33*	3	-17, -19, -22	unknown	unknown
CG8963	*mir-6*	3	-16, -17, -17	unknown	unknown
CG3638	*mir-184-3*	3	-27, -17, -22	unknown	unknown
CG33006^B^	*mir-278*	3	-20, -15, -23	unknown	unknown
CG31305^B^	*mir-278*	4	-16, -32, -29, -25	multiple	unknown
CG32206^A^	*mir-289*^A^	3	-15, -15, -16	unknown	unknown
CG12071	*mir-305*	3	-17, -21, -19	unknown	unknown

^A^: exact 5' and 3' ends for mir-263a, mir-274, mir-280, mir-282, mir-284, mir-289 have not yet been determined (6); target predictions for these microRNAs are likely to change once the precise microRNA sequences are known

^B^: gene annotated as a "gene cassette"; molecular function and biological process listed is the union of molecular functions and biological processes for each ORF in the putative dicistronic transcript

A striking feature of the set of predicted miRNA targets is the disproportionate fraction of mRNAs that encode transcription factors (13 of 41 genes with known function) or have assigned roles in neural processes (7 of 41). [41] noted a similar enrichment for transcription factors and neural genes in predicted miRNA targets in Drosophila. Because the different predictions identify groups of genes that are not fully overlapping, the bias is even more striking. The emphasis on transcription factors was also observed for predicted miRNA targets in plants [42] and mammalian cells [43].

### Validation of predicted targets

We chose a subset of three predicted miRNA-target pairs for validation in a cultured cell assay. The assay is similar to that used by Zeng and Cullen [22]. A reporter plasmid expresses, under control of the inducible metallothionein promoter [44], a hybrid mRNA in which the firefly luciferase coding region is fused to the candidate target 3' UTR (*luc/target*). The miRNA plasmid expresses, under control of the constitutive actin promoter, a genomic DNA segment that contains the miRNA primary transcript. Finally, a control plasmid expresses Renilla luciferase under control of the inducible metallothionein promoter, and serves to monitor transfection efficiency. In parallel transfections one population of S2 cells receives all of the plasmids, while a second population receives the reporter and control, but not the miRNA plasmid. Approximately 1.5 days after transfection (to allow the miRNA to accumulate), transcription of the reporter and control mRNA is induced by addition of CuSO4 to the growth medium. After an additional 6 hours, the cells are harvested and the levels of firefly and Renilla luciferase are measured.

**Figure 1:**
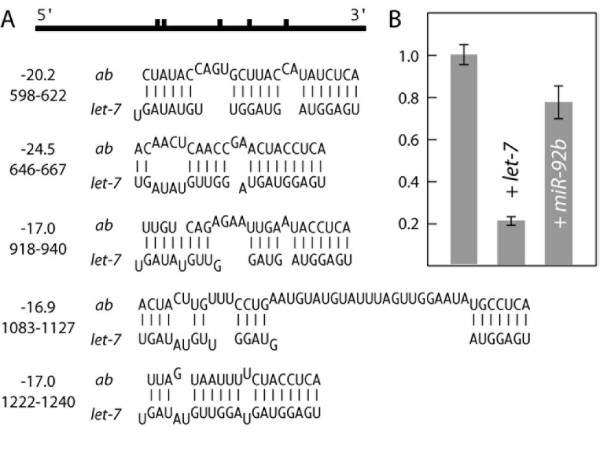
Drosophila *let-7 *miRNA targets the *abrupt *3' UTR. A. Predicted sites of *let-7 *interaction with the *ab *3' UTR. The schematic at top shows the relative positions of the sites as vertical bars in the *ab *3' UTR. The predicted pairings are shown below, with the free energies (in kcal/mol) and exact positions in the *ab *3' UTR indicated. B. Luciferase expression in transfected S2 cells. Expression from the *luciferase/ab *mRNA with no added miRNA is shown at left, set to a relative value of 1. The other bars indicate the results of coexpression with *let-7 *or *miR-92b*. In this figure and in Figure 2, all values represent the average luciferase expression from at least 5 experiments, and error bars represent standard deviation.

The *let-7 *miRNA is predicted to target the *abrupt *(*ab*) mRNA at five positions in the 3' UTR (Table 1, Fig. 1A). The level of luciferase activity from the *luc/ab *mRNA in the absence of exogenous miRNA provides the standard for measurement of the effect of the miRNA. When *let-7 *is coexpressed with the *luc/ab *reporter, the level of luciferase activity is substantially reduced (Fig. 1B). To confirm that expression of the luciferase reporter in this system is not simply sensitive to the coexpresssion of any miRNA, the experiment was also performed using *miR-92b *instead of *let-7*. Using the parameters noted above, MovingTargets does not predict *mir-92b *to target *ab*. Exogenous *miR-92b *can repress expression in the assay system of a reporter mRNA bearing the 3' UTR of a predicted *miR-92b *target (below), but has only a small effect on *luc/ab *expression, much less than the effect of *let-7 *(Fig. 1B). We conclude that *let-7 *is specifically targeting the *ab *3' UTR.

**Figure 2:**
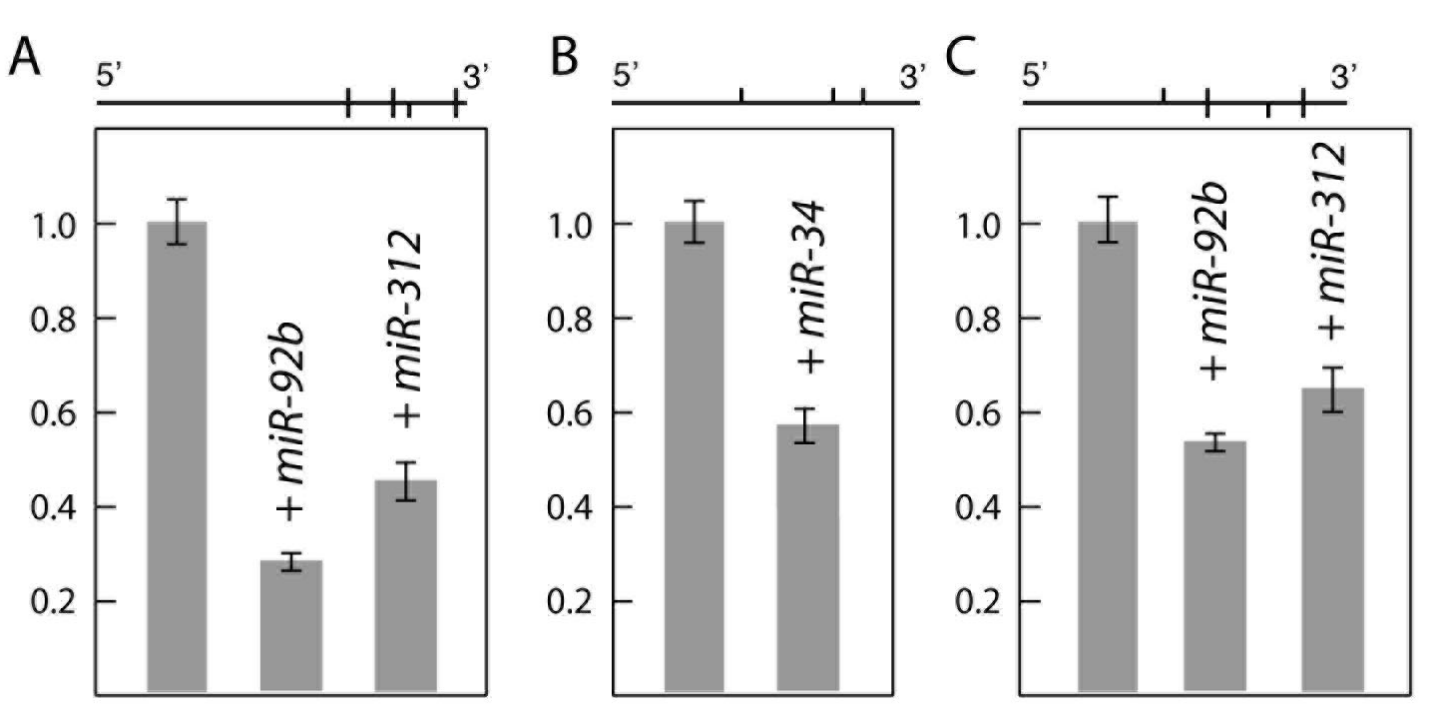
miRNA-dependent regulation of *CrebA*, *Eip74 *and *ttk*. Each panel shows at top the distribution of the miRNA target sites in the *CrebA *(A), *Eip74 *(B) and *ttk *(C) 3' UTRs. For panels A and C, the vertical bars above the line indicate sites for *miR-92b*, while vertical bars below the line indicate sites for *miR-312*. Luciferase expression data are presented as in Fig. 1.

Two additional predicted targets were tested for miRNA-dependent regulation. The *CrebA *mRNA is predicted to have three target sites for *miR-92b *and *mir-312 *(Table 1); these miRNAs are closely related, sharing the same nts in positions 2–9 and 15–21. Both miRNAs repress expression of the *luc/CrebA *reporter (Figure 2B). The *Eip74EF *mRNA is predicted to have three target sites for *miR-34 *(Table 1; Figure 2A), and *miR-34 *represses expression of the *luc/Eip74EF *reporter (Figure 2B).

### Flexible MovingTargets search to identify potential miRNAs targeting specific genes

In addition to validating a subset of our predicted targets, we wondered if we could use the flexibility of MovingTargets to identify a miRNA that regulates a gene known to be under another form of translational control. One such gene is *tramtrack *(*ttk*), which encodes a transcription factor that determines non-neuronal identity in developing sensory organ cells, and which has been shown to be translationally repressed by the RNA-binding protein Musashi [40].

*ttk *is not a predicted miRNA target using the strict biological constraints described above. However, by relaxing these constraints to require a maximum free energy of miRNA/target hybridization of -12 kcal/mole at room temperature and a minimum of 6 out of 8 consecutive base pairs in the miRNA 5' end, MovingTargets predicts that *mir-9c*, *mir-92a*, *mir-92b*, and *mir-312 *(the latter three are closely related miRNAs) target *ttk*. We tested *mir-92b *and *mir-312 *in the S2 cell assay, and both miRNAs repress expression of the *luc/ttk *reporter (Figure 2C).

## Discussion

The nature of the interaction between a miRNA and its target – incomplete and interrupted base pairing – creates a substantial challenge for the prediction of candidate miRNA targets. Furthermore, very few mRNAs have been shown to be under miRNA regulation, limiting the number of examples from which the basic rules governing miRNA/target interactions can be determined. The latter problem is particularly acute, since even in mRNAs known to be regulated, the actual target sites are usually only inferred from their partial complementarity to the miRNA. Thus a precise description of these rules is a moving target, and will undoubtedly be refined as additional targets are identified by methods not biased by current prediction strategies.

Our approach to predicting miRNA targets addresses both of these difficulties. The combination of a conserved 3' UTR database and the MovingTargets algorithm allowed us to predict 83 miRNA targets that meet stringent biological constraints based on features of the probable or proven interactions between individual miRNAs and the mRNAs under their control. Each of three target predictions chosen for testing was verified in the S2 cell transfection assay. Thus the algorithm succeeds in predicting miRNA targets. At present we have restricted the database of potential target sequences to those from 3' UTRs, but this could be expanded to include entire mRNAs given evidence that miRNAs bind to other regions of animal mRNAs. The biological relevance of a predicted miRNA/target interaction depends on whether the miRNA and target are expressed at appropriate concentrations in a particular cell type. Thus, this and all other prediction methods represent only the first step, albeit an important one, in identifying bona fide miRNA targets.

The MovingTargets software allows individual researchers to specify which constraints the software should enforce. Dramatically different predictions will result by adjusting the parameter values used here. This capacity of the software has two notable benefits. First, it provides the means to adjust the parameter values as the rules of miRNA/target interactions become better understood. Second, the adjustability of the algorithm facilitates less constrained searches that focus on a particular mRNA or miRNA. For example, if experimental evidence suggests that an mRNA is regulated by miRNAs, yet it is not among the group predicted using the stringent screening parameters, then relaxing different parameters one by one would produce a set of candidate miRNA regulators. Here the drawback of increased sensitivity – an increased number of predictions with an unknown fraction presumed to be false – would be acceptable. We were able to identify miRNAs targeting *ttk *using this strategy.

### What is the minimal quality of miRNA/target site interaction sufficient for regulation?

In the initial examples of miRNA-dependent regulation, the miRNAs very efficiently blocked accumulation of the proteins encoded by the target mRNAs. These dramatic effects could be the normal mode of miRNA-dependent regulation: when some quality of miRNA/mRNA target interaction occurs, then protein accumulation is blocked. Alternatively, the degree of regulation could be directly correlated with the quality of the individual interactions (or their sum), and several mutation studies have shown that the level of translational repression of a miRNA target varies in relation to the quality of miRNA targeting, as defined by such characteristics as free energy of hybridization, consecutive 5' base pairing, and number of target sites [3,22,39]. In our experiments to validate the predicted miRNA targets we tested a miRNA, *miR-92b*, not predicted to interact with the *ab *3' UTR. Although *miR-92b *did not produce the strong inhibitory effect of *let-7*, it did cause some inhibition. One interpretation is that *miR-92b *weakly interacts with the *ab *3' UTR, and that the weak interaction is sufficient for a weak regulatory effect. When the 'number of target sites' parameter of MovingTargets was relaxed to two, rather than three, we found that *miR-92b *is predicted to target *ab *at two sites that satisfy the remaining strict biological constraints. Thus our results are also consistent with the notion that there is a correlation between the overall strength of miRNA/mRNA interactions and the degree of regulation. An implication of this conclusion is that even weak interactions may have consequences, and that many or even most of all cellular mRNAs may be regulated, but that the degree of regulation may vary substantially.

### Comparison of different prediction results

The high-likelihood miRNA targets predicted in this paper using strict biological constraints produce very different results in comparison to the other Drosophila miRNA target prediction algorithms. The simplest forms of comparisons are not possible, because the other predictions produce a ranked list of predicted targets for each miRNA, while our method identifies a group of miRNA targets that adhere to specific constraints. Nevertheless, when considering our group of 83 predicted miRNA targets, only 11 are included among the top 20 targets for any individual miRNA predicted by Enright et al. [41], only 13 appear in the top 50 targets for individual miRNAs predicted by Stark et al. [45], and only 4 of the 83 are predicted by Rehmsmeier et al. [46].

There are several possible reasons for the differences in the predictions by the different algorithms. First, only our approach goes beyond thermodynamic stability considerations in imposing a penalty on G:U base pairs involving the 5' part of the miRNA. Second, the value of multiple target sites is treated differently (there is experimental evidence of synergy between multiple target sites and most known miRNA targets have multiple predicted target sites [2,21,23-28]. For example, Enright et al. reward for multiple sites by summing a score for all complementary sites in the target 3' UTR, whereas our algorithm requires a miRNA/target pair to have a user-specified absolute number of target sites each meeting a user-defined set of biological constraints. A third difference centers on the importance of extensive base pairing in the miRNA 5' region, for which there is both experimental evidence and the precedent of the predicted interactions between known target mRNAs and their miRNAs [2,23-28]. Our algorithm requires a miRNA-target interaction to have a user-specified minimum absolute number of consecutive and total base pairs in the miRNA 5' region. In contrast, Enright et. al. appear not to heavily weight this feature, since many of their top-rated miRNA/target interactions have significant gaps in the miRNA 5' region.

Five other miRNA target prediction methods for animals have been published, but the predictions cannot be compared directly to ours since four of the five examined mammalian mRNAs and the other tested only a small number of Drosophila genes [38,43,47-49]. None of these approaches is identical to ours, and so if used with Drosophila mRNAs and miRNAs, each would be expected to provide results not identical with ours, just as for the published examples.

## Conclusion

Prediction of animal miRNA targets is a challenging task due to the incomplete and interrupted base pairing between a miRNA and its target. We developed the MovingTargets software program to provide a tool for the accurate and flexible prediction of miRNA targets in Drosophila. Using this tool, we identified a set of 83 high-likelihood miRNA targets. We tested and verified 3 of these predictions, including a target for the Drosophila *let-7 *homolog.

MovingTargets provides flexibility in describing the characteristics defining a miRNA target. Thus, as the rules governing miRNA-target interactions are better elucidated, these constraints can be enforced through MovingTargets to produce more refined sets of miRNA target predictions. We used this flexibility to relax the constraints placed on a miRNA-target interaction to predict and validate miRNAs targeting *tramtrack*. MovingTargets is freely available on DVD by request.

## Availability and Requirements

The MovingTargets software is available on DVD by request. It can be used on any Perl platform, such as the Macintosh 'Terminal' utility. Usage of the software requires only minimal computer skills. The researcher can specify the biological constraints for a miRNA target search through the user interface. In addition, the researcher can specify a single target for focused searches with individual mRNAs. For a miRNA target search of the entire target database, the program runs in about 2 hours on an earlier generation Macintosh computer (466 MHz G4); focused searches for miRNAs targeting an individual mRNA are much faster and take about 20 minutes.

## List of abbreviations

miRNA microRNA

UTR untranslated region

RNAi RNA interference

RISC RNA-induced silencing complex

PCR polymerase chain reaction

DNA deoxyribonucleic acid

RT room temperature

FBS fetal bovine serum

D. melanogaster Drosophila melanogaster

D. pseudoobscura Drosophila pseudoobscura

BDGP Berkeley Drosophila Genome Project

mRNA messenger RNA

## Authors' contributions

CB designed and implemented the software, performed all of the experiments and drafted the manuscript. CB and PMM together conceived of the study, and PMM helped to draft the manuscript
